# Charge-Flow Profiles along Curvilinear Paths: A Flexible Scheme for the Analysis of Charge Displacement upon Intermolecular Interactions

**DOI:** 10.3390/molecules26216409

**Published:** 2021-10-23

**Authors:** Luca Sagresti, Sergio Rampino

**Affiliations:** 1 Scuola Normale Superiore, Piazza dei Cavalieri 7, 56126 Pisa, Italy; luca.sagresti@sns.it; 2 Istituto Nazionale di Fisica Nucleare (INFN)—Sezione di Pisa, Largo Bruno Pontecorvo 3, 56127 Pisa, Italy

**Keywords:** electron density, chemical bonding, intermolecular interactions, charge displacement, curvilinear interaction paths

## Abstract

The Charge-Displacement (CD) analysis has proven to be a powerful tool for a quantitative characterization of the electron-density flow occurring upon chemical bonding along a suitably chosen interaction axis. In several classes of interesting intermolecular interactions, however, an interaction axis cannot be straightforwardly defined, and the CD analysis loses consistency and usefulness. In this article, we propose a general, flexible reformulation of the CD analysis capable of providing a quantitative view of the charge displacement along custom curvilinear paths. The new scheme naturally reduces to ordinary CD analysis if the path is chosen to be a straight line. An implementation based on a discrete sampling of the electron densities and a Voronoi space partitioning is described and shown in action on two test cases of a metal-carbonyl and a pyridine-ammonia complex.

## 1. Introduction

Since Lewis’ original work on electron-pair sharing in 1916 [1], chemical bonding is usefully described in terms of the associated electron-charge redistribution. While for decades the movements of electrons between interacting partners have been—and still schematically are—represented by dots and lines through the popular Lewis’ diagrams, the advent of modern electronic-structure methods coupled to the computer revolution has made it possible to compute such electron-charge redistributions within a quantum-mechanical framework. In fact, a static picture of the electron-charge redistribution occurring upon chemical bonding can be conveniently obtained by computing a suitably formulated electron-density difference between an adduct and its constituting fragments, thus obtaining a three-dimensional function Δρ(x,y,z) being negative in regions of electron depletion and positive in regions of electron accumulation. As shown by the pioneering works of Bader on diatomics in his late-1960s works [2], a deep insight into the nature of chemical bonding can indeed be gained through a careful analysis of the topological features of Δρ(x,y,z).

Chemical-bond analysis can nowadays rely on a broad class of methods (see, for instance, Reference [3]) rooted in quantum mechanics, some of which focus on the energy partitioning (SAPT [4,5], EDA [6]), some on the topology of the molecular electron density (QTAIM [7]), and some others on the wavefunction itself (ELF [8], ELI-D [9]). Among the available methods, the recently proposed Charge–Displacement (CD) analysis [10] targets the above-mentioned electron-density redistribution Δρ(x,y,z) directly and aims at extracting a quantitative picture of the charge flow occurring upon bond formation along a directional axis (usually indicated with *z* and chosen so as to represent the interaction axis) through the one-dimensional CD function

(1) $$\Delta q\left(z\right)=\int_{-\infty }^{z}dz^{\prime }\int_{-\infty }^{\infty }\int_{-\infty }^{\infty }\Delta \rho \left(x,y,z^{\prime }\right)\;dx\;dy\;,$$

providing a *z* resolved clear-cut measure of the intra- and inter-fragment charge transfer associated with the intermolecular interaction. In spite of its simple formulation, the CD analysis has proven to be a powerful instrument capable of casting light on controversial chemical interactions (see for instance References [11,12,13]) and plays a key role in the recently devised composite Natural Orbitals for Chemical Valence/Charge Displacement (NOCV/CD) [14,15] analysis scheme. The NOCV/CD scheme allows one to compute the overall Δρ associated with the formation of a chemical bond between two partners as well as its components in terms of molecular orbitals, and to extract from them a quantitative charge-flow profile along the interaction axis *z* by means of the above simple integration. Such scheme has been successfully used to quantitatively analyze the coordination bond in organometallic complexes in terms of its σ-donation and π-backdonation components [16,17,18], to address other challenging interactions [19,20,21], and to aid in the interpretation of more qualitative models such as the resonance structures [22]. Moreover, a relativistic version of the analysis scheme has been formulated [23,24] and ported to a fully relativistic four-component electronic-structure program [25,26].

As a matter of fact, the CD function of Equation (1) is a simple and powerful tool for analyzing on quantitative grounds the displacement of the electrons associated with an intermolecular interaction when an interaction axis *z* can be identified. However, the usefulness and consistency of the method becomes much more questionable when the charge flow associated with the interaction at hand does not occur along a straight line and a univocal interaction axis cannot be defined. A few notable cases of such interactions are, for example, the heme group (or similar porphyrin complexes) binding molecular oxygen, cyanide or carbon monoxide, where the interaction develops along a right angle; amines attacked by an electcrophile, where the charge flow develops along the typical tetrahedral angle; or even more trivially, hydrogen bonding in water.

The scope of the present article is to define a general and flexible formulation of the CD analysis capable of accounting for non-linear interaction paths such as those mentioned above and reducing to the original one when the path is chosen to be linear. Accordingly, the article is organized as follows. In Section 2, the new formulation is discussed with reference to custom curvilinear paths. In Section 3, computational details for a practical implementation are given. In Section 4, the method is tested and shown in action on two molecular complexes. In Section 5, conclusions are drawn and perspectives are outlined.

## 2. Methodology

As already mentioned, the CD analysis through the CD function of Equation (1) holds when the interaction develops along a straight directional axis. This can either be one of the reference axes or any other straight line that can be realigned along the *z* axis by rotation of the system and of the associated Δρ(x,y,z). The evaluation of the CD function can, in this case, be made in a two-step procedure. First, a one-dimensional profile of Δρ(x,y,z) along the *z* axis is computed by integration over the xy planes

(2) $$\Delta \rho \left(z\right)=\int_{-\infty }^{\infty }\int_{-\infty }^{\infty }\Delta \rho \left(x,y,z\right)\;dx\;dy\;,$$

returning for each *z* an ‘xy-averaged’ picture of the electron loss/gain. Then, this quantity can be converted to the CD function by progressive integration:

(3) $$\Delta q\left(z\right)=\int_{-\infty }^{z}\Delta \rho \left(z^{\prime }\right)dz^{\prime }\;.$$

Compared to Δρ(z), the CD function conveys the same information in a more advantageous way, in that for each *z* point it coincides with the amount of electron charge that, upon formation of the bond, has crossed from right to left (the direction of decreasing *z*) a plane orthogonal to the *z* axis through that point. It should be noted that, due to the definition of Δρ(x,y,z), the Δρ(z) and the Δq(z) functions include both the charge transfer between the interacting partners and the charge displacement due to intra-fragment reorganization of the electron cloud. On the other hand, as discussed in Reference [14], a clear-cut estimate of the charge transfer between the interacting partners can be easily extracted from the CD function by taking its value at a suitably defined, so-called ‘isoboundary’, *z* point in the axis segment between the fragments.

What we seek in this article is a general and flexible formulation of the CD analysis for an arbitrary curvilinear path c(x,y,z) in three-dimensional Euclidean space, including, as a special case, the straight paths used in ordinary CD analysis. In other words, in analogy with Equation (2), we need to formulate a one-dimensional Δρ(c) that will have the form:

(4) $$\Delta \rho \left(c\right)=\int_{S}\Delta \rho \left(x,y,z\right)\;dS\;,$$

with *S* being a *c*-dependent suitably defined surface, and that, in analogy with Equation (5), can be easily integrated to give the one-dimensional CD function along *c*:

(5) $$\Delta q\left(c\right)=\int_{-\infty }^{c}\Delta \rho \left(c^{\prime }\right)\;dc^{\prime }\;.$$

It is clear that the core problem is that of finding a suitable definition of the *c*-dependent integration surfaces *S* that have to span the whole three-dimensional space and ensure the correct counting of the electrons in the molecule. This can be easily achieved through a Voronoi scheme for space partitioning [27], whereby, for a given point *c*, *S* identifies the sets of points in three-dimensional space, which are closer to point *c* than to any other point of the curvilinear path. It can be easily verified that if the curvilinear path is chosen as a straight line coinciding with the *z* axis, then *S* coincides with the xy plane orthogonal to the *z* axis through a given *z* point and Equation (4) reduces to Equation (2).

As any practical implementation has to resort to discrete representations of Δρ(x,y,z), we switch now to the discrete version of Equations (2)–(5). Many electronic-structure programs, in fact, are capable of computing Δρ(x,y,z) and save it as a discretized function over a user-defined $N_{x}\times N_{y}\times N_{z}$ three-dimensional grid with constant spacings Δx, Δy, and Δz according to the popular .cube file format. Now, if we write the fraction of electron loss/gain contained in a cell of the discretization grid as $\Delta \rho_{ijk}=\Delta \rho \left(x_{i},y_{i},z_{k}\right)\Delta x\Delta y\Delta z$, the discrete versions of Equations (2) and (3) are

(6) $$\Delta \rho \left(z_{k}\right)=\frac{1}{\Delta z}\sum_{i=1}^{N_{x}}\sum_{j=1}^{N_{y}}\Delta \rho_{ijk}$$

and

(7) $$\Delta q\left(z_{k}\right)=\sum_{k^{\prime }=1}^{k}\Delta \rho \left(z_{k^{\prime }}\right)\Delta z\;,$$

where the summations in Equation (6) run over the number of points $N_{x}$ and $N_{y}$ along the *x* and *y* directions, respectively, of the discretization grid.

By discretizing as well the curvilinear path with constant spacing Δc and indicating its points with $c_{l}$, the discrete analogue of Equations (4) and (5) become:

(8) $$\Delta \rho \left(c_{l}\right)=\frac{1}{\Delta c}\sum_{ijk\in S_{l}}\Delta \rho_{ijk}$$

and

(9) $$\Delta q\left(c_{l}\right)=\sum_{l^{\prime }=1}^{l}\Delta \rho \left(c_{l^{\prime }}\right)\Delta c\;,$$

where in Equation (8), the dependence of *S* on *c* has been made explicit by the subscript *l*, and the summation includes, for a given point $c_{l}$, all the points belonging to the discrete version of $S_{l}$, i.e., all the points $\Delta \rho_{ijk}$ closer to the actual $c_{l}$ than to any other point of the path. Note that a point $\Delta \rho_{ijk}$ may actually feature an equal minimum distance with more, say *m*, than one of the path points. In this case, a viable solution to ensure a proper counting of the total electron-charge loss/gain is to assign a value of charge loss/gain equal to $\Delta \rho_{ijk}/m$ to each of the *m* points.

## 3. Path Generation and Voronoi Tessellation

As evident from the discussion in the previous sections, the novel aspects in the curvilinear CD analysis are the generation of a curvilinear path c(x,y,z) and the calculation of the intermediate quantity Δρ(c) by integration over (discretized) curved surfaces rather than over xy planes. In this section, we give some details on a practical implementation of the method with a focus on these two aspects. While the methodology outlined in the previous section holds for any discrete curvilinear path described by a sequence of equally-distanced (x,y,z) points, for the purpose of the present work we will consider curvilinear paths generated as polygonal chains connecting a list of user-selected atoms. A Fortran 95 program implementing such curvilinear CD analysis is available upon request to the authors.

The program requires (i) a .cube file containing the geometry of the molecular system under study and the definition of the discretization grid, (ii) an ordered list of atom IDs specifying the atoms to be connected by the path, and (iii) a discretization step Δc. The program identifies the segment connecting the first and the second atom of the list, and then extends such segment in the direction from the second to the first atom until the wall of the discretization box is reached. The path *c* starts from the intersection of the extended segment and the wall of the box and follows the straight line passing through the first and the second atom. Once this has been reached, the path changes direction and points towards atom three, and so on until the last atom is reached and the path is prolonged along the straight line connecting the two atoms of the list until one of the walls of the discretization box is reached. The entire protocol has been coded in such a way that during the definition of the discretized points of the curvilinear path, a constant increment Δc is adopted. The path is printed in an output file containing the number of points in which the path has been discretized, the discretization step Δc, and the list of path points as x,y,z triplets.

The integration procedure involved in the computation of Δρ(c) (Equation (4)) is performed according to the formalism discussed in the previous section. In particular, our program reads as input the generated path and the .cube file containing the electron density redistribution Δρ(x,y,z). Then, for each point of the three-dimensional grid, the distance between that point and the points of the path is scanned, and the associated fraction of electron loss/gain $\Delta \rho_{ijk}$ is assigned to the closest point(s) as discussed in Section 2. Once the Δρ(c) and Δq(c) functions have been computed, they are printed as four-column files containing a list of x,y,z triplets corresponding to the path points, and the associated value of the function. This format has been chosen as it is easily read by a simple Python script allowing for the effective graphic representations presented in the next section.

## 4. Applications

In order to assess the features of the newly developed methodology, we have tested it on two molecular systems, the metal carbonyl complex [CuCO]${}^{+}$ and the pyridine–ammonia complex. The rationale for the choice of this pair of systems has been the following: the first system was chosen to test the novel formulation of the CD analysis and validate it against the original one, the second system was chosen so as to demonstrate and highlight the potentialities of the novel methodology with respect to the original one. In particular, in the [CuCO]${}^{+}$ case, we focus on the charge redistribution associated with the formation of the copper–carbon bond from fragments Cu${}^{+}$ and CO. As already mentioned, this is a simple linear complex that can be effectively analyzed with ordinary CD analysis and will thus serve as a benchmark to test and validate the curvilinear formulation in order to assess its consistency. The second is a more challenging case of a complex of pyridine and ammonia where we focus on a σ-type interaction involving both a N–H⋯N and C–H⋯N hydrogen bond [28]. It should be stressed here that this pair of systems was chosen for the purpose of validating and demonstrating the potentialities of the newly developed algorithm. Accordingly, a detailed study of the intermolecular interaction in these and other more complex applications is out of the scope of the present paper. As outlined in the conclusions, work is ongoing in our laboratory in this direction on several challenging molecular systems.

### 4.1. Computational Details

Both the considered examples were taken from available literature (see Reference [15] for [CuCO]${}^{+}$ and Reference [28] for the pyridine–ammonia complex). In this work, for the sake of consistency, we adopted the same level of theory used in the original references for their quantum-chemistry characterization. For the reader’s convenience, the computational details adopted therein and in the present work are summarized in the following, while the reader is referred to the original references for further details. Optimized structures and electron densities were obtained in vacuo by Density Functional Theory (DFT) using the Gaussian suite of programs (G16) [29]. For [CuCO]${}^{+}$, the B3LYP [30,31] exchange-correlation functional was adopted in conjunction with a LANL2DZ basis set with effective core potential for copper [32] and a 6–31+G* [33,34,35] basis set for the other atoms. Note that alternative exchange-correlation functionals and an all-electron basis set could have been easily adopted for this system. However, for the purpose of validating the methodology presented in this article against published results, we decided to adopt the same established computational protocol already used in previous works on bond analysis [15,17,18,22]. For the pyridine-ammonia complex, the double-hybrid B2PLYP [36] exchange-correlation functional was adopted in conjunction with the triple-ζ*m*-aug-cc-pVTZ basis set [37] in which *d* functions on hydrogen atoms were removed. In all cases, semi-empirical dispersion contributions were taken into account by inclusion of the Grimme’s D3BJ [38] model as implemented in the Gaussian software.

For each system, the electron-density difference Δρ(x,y,z) was computed as the difference between the electron density of the adduct and those of the two isolated fragments frozen at their in-adduct geometries. The two charge redistributions Δρ(x,y,z) were discretized on a three-dimensional grid defined by adding a margin of 5 Bohr to each of the *x*, *y*, *z* extremes of the molecular geometry with constants spacings Δx=0.2 Bohr, Δy=0.2 Bohr, Δz=0.1 Bohr.

### 4.2. [CuCO]${}^{+}$

**Figure 1:**
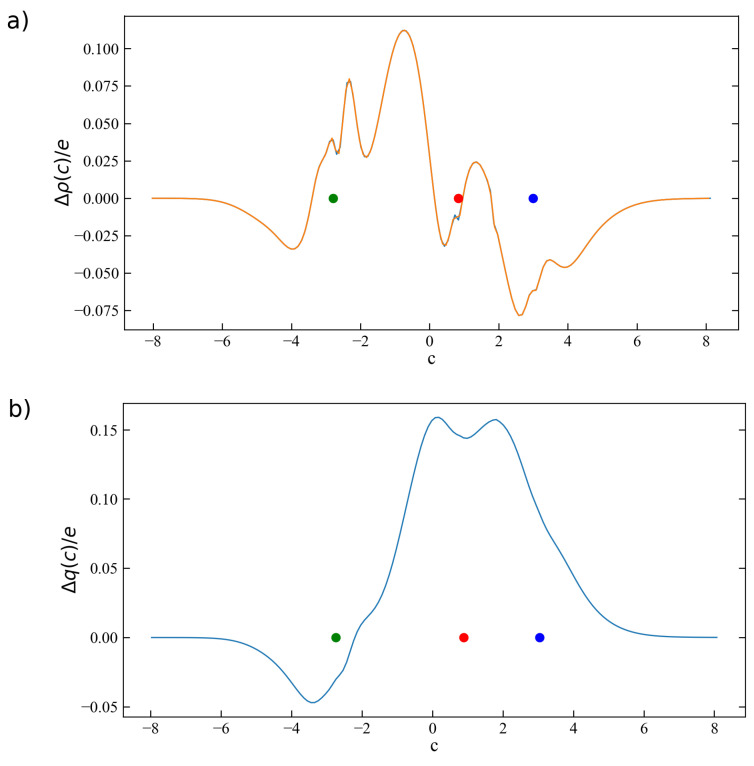
(**a**) Δρ(c) from curvilinear CD analysis (blue curve) along a linear *c* path superimposed to the *z* axis. Δρ(z) from ordinary CD analysis (orange curve) is also shown. (**b**) Δq(c) from curvilinear CD analysis (blue curve). The positions of the copper, carbon, and oxygen nuclei along the axis are indicated in green, red, and blue, respectively.

The charge-redistribution profile Δρ(c) and the CD function Δq(c) along the curvilinear path *c* purposely chosen as a straight path passing through the three atoms lying on the *z* axis and discretized with a constant step of Δc=0.1 Bohr are shown in Figure 1.

In panel (a), the charge–redistribution profile Δρ(z) obtained by ordinary CD analysis (Equation (2)) is also shown as the orange curve. As evident, Δρ(c) and Δρ(z) are in very good agreement, with barely perceptible differences due to the fact that, while Δc has been chosen to be equal to Δz, the sampled points along the two paths *c* and *z* do not necessarily match and the two sets of points are in fact slightly shifted with respect to each other.

A comment is due here on the choice of Δc. For the purpose of comparing the curvilinear versus ordinary CD analysis, the most obvious choice was that of setting Δc equal to the Δz of the discretization grid that is also used in ordinary CD analysis. Generally speaking, a different value for Δc could have been chosen. However, particular care has to be put in this choice because too large a Δc would return a very approximate and poorly sampled Δρ(c) profile, while too small a Δc would lead to an over-sampled Δρ(c), featuring some oscillations due to points of the path to which no fraction of electron loss/gain was assigned on the basis of the proximity criterion discussed in Section 2. As a rule of thumb, a Δc comparable with the spacings used for the grid definition (Δx, Δy, and Δz) is a good choice. On the other hand, as will be clear from the forthcoming discussion on the second considered molecular system, this issue is far less relevant in the calculation of Δq(c) due to the peculiar definition of this function as a progressive integration.

The CD function Δq(c) for the copper–carbon interaction in the [CuCO]${}^{+}$ complex is shown as a blue curve in panel (b) of Figure 1. The curve is positive almost everywhere along the interaction axis, with the exception of a small negative peak on the rear side of the copper atom. This means that in the region of the interaction between copper and carbon there is a net charge transfer (with a maximum value of about 0.16 *e*) in the direction from carbon to copper. On the other hand, the negative values of Δq(c) in the proximity of the copper atom indicate that an intra-fragment charge redistribution is occurring in the opposite direction, i.e., from left to right (with a minimum value of 0.05 *e*). The CD curve is also positive in the region between the C and O atoms, indicating that a polarization of the electron cloud in the C←O direction is also occurring upon bond formation. These features have been thoroughly analyzed and interpreted on the basis of the σ-donation and π-backdonation components of the coordination bond through a NOCV/CD analysis, and the interested reader is referred to Reference [15] for a more detailed discussion.

### 4.3. Pyridine–Ammonia Complex

**Figure 2:**
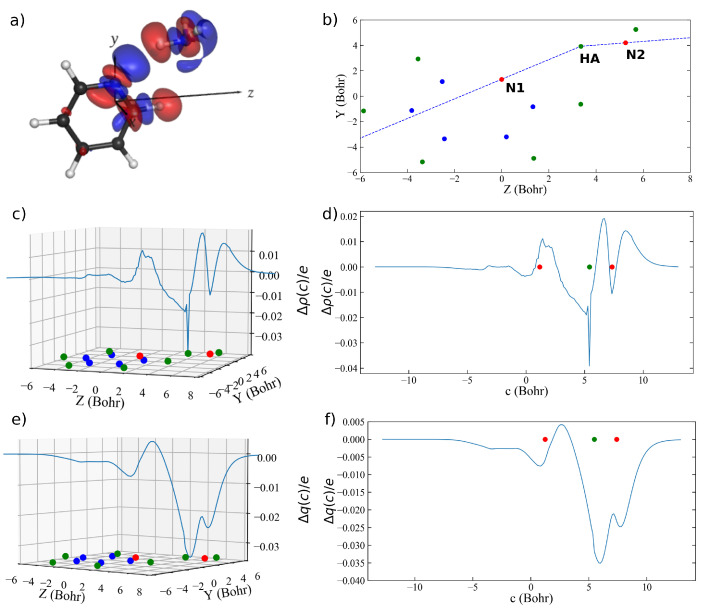
(**a**) Δρ(x,y,z) for the pyridine–ammonia complex (isodensity surfaces at ±0.0005 e/bohr${}^{3}$; red lobes: electron depletion; blue lobes: electron accumulation). (**b**) Curvilinear path *c* lying in the pyridine molecular plane. (**c**) Δρ(z) represented on the pyridine molecular plane. (**d**) Δρ(z) linearized as a function of the curvilinear coordinate *c*. (**e**) Δq(z) represented on the pyridine molecular plane. (**f**) Δq(z) linearized as a function of the curvilinear coordinate *c*. The position of the hydrogen, carbon, and nitrogen atoms are indicated with green, blue, and red dots on the molecular plane, respectively.

As already mentioned, the second considered case is a pyridine–ammonia complex resulting from a σ-type interaction involving multiple concurrent charge flows [28]. In fact, as shown by the isodensity surfaces in panel (a) of Figure 2, the charge redistribution associated with the pyridine–ammonia interaction involves two charge-flow channels: the first one extends from the pyridine N atom to the ammonia fragment and is associated with the N–H⋯N hydrogen bond; the second one develops along the C–H bond of one of the C atoms adjacent to the pyridine N atom and runs almost in parallel with respect to the first one. This second charge flow is in fact associated with an additional C–H⋯N (with N being the ammonia N atom) hydrogen bond.

When approaching this system from the perspective of ordinary CD analysis, a natural choice for the integration axis *z* would be the axis passing through the two N atoms involved in the main interaction between the two fragments. However, as evident from the isodensity surfaces in panel (a) of Figure 2, due to the second charge flow developing in an almost parallel direction, the resulting charge–redistribution profile Δρ(z) and CD function Δq(z) would return an averaged picture of the charge displacement due to both interactions and it would be impossible to discern the specific features of each of them. Moreover, focusing again on the first charge-flow channel associated with the N–H⋯N hydrogen bond, one can easily see by inspection of the isodensity surfaces of Δρ(x,y,z) that the charge flow develops along a polygonal chain connecting the three involved atoms rather than along a straight line connecting the two N atoms.

A first improvement that can be achieved through curvilinear CD analysis is thus that of defining an interaction path that connects the pyridine N to the ammonia H atom, and then this atom to the ammonia N atom. Accordingly, following the procedure described in Section 3, we generated the path shown in panel (b) of Figure 2.

The charge–flow profile Δρ(c) along this path resulting from curvilinear CD analysis using a Δc of 0.125 Bohr is shown as a blue curve in a three-dimensional plot as a function of the molecular plane in panel (c) of Figure 2, and linearized in panel (d) of the same figure as a function of the curvilinear coordinate *c*. The slight oscillations observed in the Δρ(c) curve are, as previously mentioned, due to the discrete nature of the data to be integrated and to the resulting inherently approximate Voronoi tessellation. These oscillations could in principle be reduced by fine tuning of the ratio between the discretization grain of the electron densities and the choice of Δc. However, as already mentioned, they turn out to be of very low relevance when moving from Δρ(c) to Δq(c), as the latter is a much smoother function due to its peculiar formulation as a progressive integration.

The CD function Δq(c) for the pyridine–ammonia interaction along the curvilinear path *c* is shown as a blue curve in a three-dimensional plot as a function of the molecular plane in panel (e) of Figure 2, and linearized in panel (f) as a function of the curvilinear coordinate *c*. As evident from the plot, Δq(c) is negative almost anywhere along *c*, indicating a charge flow in the left to right direction along the whole curvilinear path with the exception of a small segment between the pyridine N atom and the ammonia H atom where the charge flow occurs in the opposite direction (positive values of Δq(c)). The charge transfer from pyridine to ammonia upon their interaction, i.e., the charge transfer from left to right along *c*, reaches a maximum (corresponding to the absolute minimum of Δq(c)) of 35 m*e* in the proximity of the ammonia H atom.

**Figure 3:**
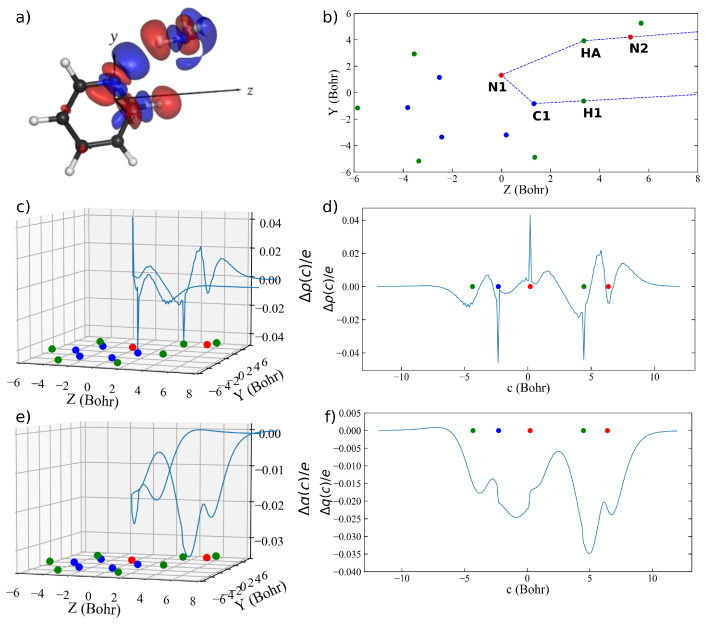
(**a**) Δρ(x,y,z) for the pyridine–ammonia complex (isodensity surfaces at ±0.0005 e/bohr${}^{3}$; red lobes: electron depletion; blue lobes: electron accumulation). (**b**) Curvilinear path *c* lying in the pyridine molecular plane. (**c**) Δρ(z) represented on the pyridine molecular plane. (**d**) Δρ(z) linearized as a function of the curvilinear coordinate *c*. (**e**) Δq(z) represented on the pyridine molecular plane. (**f**) Δq(z) linearized as a function of the curvilinear coordinate *c*. The position of the hydrogen, carbon, and nitrogen atoms are indicated with green, blue, and red dots on the molecular plane, respectively.

However, for the same reasons mentioned when discussing the shortcomings of the ordinary CD analysis applied to this complex, also a curvilinear CD analysis conducted along the N–H⋯N interaction path would unfortunately fail to properly characterize the two different charge flows that are clearly visible by inspection of the three-dimensional Δρ(x,y,z). Therefore, in order to obtain a resolved picture including the overall charge displacement occurring upon formation of the complex, we generated a new, more appropriate path shown in panel (b) of Figure 3. Such path follows the peculiar geometry of the overall charge flow suggested by inspection of the three-dimensional isodensity surfaces, which appears to develop along a polygonal chain connecting (see panel (b) of Figure 3 for the atom labels) H1 to C1, C1 to N1, N1 to HA, and HA to N2.

The charge-flow profile Δρ(c) along this path resulting from curvilinear CD analysis using a Δc of 0.1 Bohr is shown as a blue curve in a three-dimensional plot as a function of the molecular plane in panel (c) of Figure 3, and linearized in panel (d) of the same figure as a function of the curvilinear coordinate *c*. Again, as already mentioned, the oscillations observed in the Δρ(c) curve are due to the discrete nature of the data to be integrated and to the resulting inherently approximate Voronoi tessellation, and are of no relevance when moving from Δρ(c) to Δq(c).

The CD function Δq(c) along the curvilinear path *c* is shown as a blue curve in a three-dimensional plot as a function of the molecular plane in panel (e) of Figure 3, and linearized in panel (f) as a function of the linear coordinate *c*. The right-hand-side end of the curve well overlaps with the previously discussed curve of Figure 2, panel (f). However, the middle and left-hand-side regions are remarkably different. In particular, in the region between the pyridine N and the ammonia H atoms, the positive values featured by Δq(c) along the previously considered path following the only N–H⋯N interaction (Figure 2) find no place here. This means that the previously discussed positive peak featured in panel (f) of Figure 2 is actually an artifice resulting from the ‘noise’ generated by the second charge-flow channel running almost parallel to the first one. When adopting the path of Figure 3, instead, the charge flow turns out to be entirely mono-directional along the whole path between H1 and N2 and features two additional relative minima of 18 m*e* between H1 and C1, and of 25 m*e* between C1 and N1, allowing for a detailed characterization of both the concurrent charge-flow channels simultaneously.

## 5. Conclusions

The Charge–Displacement (CD) analysis is a simple and powerful tool for analyzing on quantitative grounds the features of the charge redistribution occurring upon intermolecular interactions along a selected directional axis. As discussed in the article, however, in several interesting molecular systems, the interaction does not develop along a straightforward directional axis but can follow more complex paths in three-dimensional space. In these cases, the application of the ordinary CD analysis becomes troublesome and may lead to misleading or incorrect results.

This prompted us to define a general and flexible formulation of the CD analysis capable of overcoming this issue by returning charge–flow profiles along custom curvilinear paths in three-dimensional space. The curvilinear paths can be chosen as polygonal chains connecting an ordered list of atoms, made to coincide with the QTAIM bond paths or defined as totally custom ones. The developed curvilinear CD scheme naturally reduces to ordinary CD analysis if the path is chosen to be a straight line. An implementation of the methodology based on a Voronoi tessellation of discretized electron-density differences was presented and shown in action on two molecular systems.

Calculations were presented on a pair of test systems in order to (i) validate the newly developed algorithm against the original one and (ii) demonstrate its potentialities. The results prove the consistency of the new scheme with respect to the original one, and highlight the advantages of the new scheme in properly catching features of complex intermolecular interactions which are completely missed by the old scheme. This prompts for future work on more complex systems such as metal–carbene complexes, weak non-covalent interactions including halogen bonding and π-π stacking, and systems with intramolecular hydrogen bonding, which will be the subject of forthcoming publications.

This new, flexible reformulation empowers CD analysis retaining all of its features and extending its applicability to cases where a straightforward interaction axis cannot be defined or is not the most suitable choice. The scheme can be at no additional cost integrated into the powerful composite NOCV/CD analysis, allowing for a resolution of the curvilinear charge flows in terms of the relevant molecular orbitals and permitting, for instance, to track the σ-donation and π-backdonation charge flows along curvilinear paths in complex coordination compounds. Furthermore, beyond the domain of intermolecular interactions, it can also be conveniently adopted for analyzing quantitatively the charge flows associated with electronic excitations. Work is ongoing in our group in these directions and towards the integration of this methodology with the recently developed virtual laboratory for the analysis of chemical bonding [15,39] and with more affordable immersive-virtual-reality technologies such as the head-mounted displays [40].
